# Genome-wide analysis of *KCS* gene family in sunflower reveals the involvement of *HaKCS1-1* in wax biosynthesis and drought resistance

**DOI:** 10.1186/s12870-026-09395-9

**Published:** 2026-07-02

**Authors:** Yunneng Wei, Jiabao Yang, Xiuli Yang, Jingbo Dang, Li Sun

**Affiliations:** College of Life Sciences, Shihezi University, Shihezi, Xinjiang 832003 China

**Keywords:** Sunflower, *KCS* gene family, Cuticular wax, Drought stress response, Transgenic *Arabidopsis*

## Abstract

**Background:**

The β-ketoacyl-CoA synthase (KCS) family is responsible for synthesizing very-long-chain fatty acids (VLCFAs), which are precursors of cuticular waxes and plant responses to environmental stimuli. However, knowledge of this family in sunflower (*Helianthus annuus* L.) remain limited.

**Results:**

In this study, 26 KCS family members were identified in the sunflower genome, which were unevenly distributed over 16 of the 17 chromosomes. The HaKCS proteins were divided into eight subclasses based on their phylogenetic analysis: α, β, γ, δ, ζ, ε, η, and θ. Promoter analysis indicated that *HaKCS* genes contain a number of *cis*-elements associated with stress and hormone responses. Expression profiling revealed that *HaKCS* genes were highly expressed in the stems, leaves and cotyledons of sunflower. Furthermore, most of *HaKCS* genes were up-regulated in response to exogenous abscisic acid (ABA) and drought stress. Functional study revealed that *HaKCS1-1* heterologous expression in the *Arabidopsis kcs1* mutant raised the accumulation of fatty acid (C20:0), alkanes (C29:0, C31:0, and C43:0), and alcohol (C26:0) in stems. It also improved drought tolerance, reduced leaf epidermal permeability, and alters the morphology of cuticular wax crystals on the stems of transgenic *Arabidopsis*.

**Conclusion:**

In summary, 26 *KCS* genes were identified in sunflower and exhibited stress-responsive expression patterns. *HaKCS1-1* alters the crystal structure or morphology of cuticular wax, lowering epidermal permeability and increasing drought tolerance in transgenic *Arabidopsis*. These findings shed light on the role of *KCS* genes in stress adaptation and provided candidate genes for molecular enhancement of wax content in sunflower.

**Supplementary Information:**

The online version contains supplementary material available at https://doi.org/10.1186/s12870-026-09395-9.

## Introduction

The surfaces of aerial plants are covered by the cuticle, a specialized hydrophobic layer that acts as the primary interface between the plant and its surroundings. This layer is primarily composed of a cutin polymer matrix and cuticular waxes, which are further categorized into intracuticular and epicuticular waxes, and epicuticular wax is deposited on the outermost surface, often self-assembles into various microscopic crystalline structures like rods, tubes, and plates [1]. As the first line of defense of plants, cuticular waxes play a crucial role in protecting plants against biotic and abiotic stresses by reducing non-stomatal water loss, enhancing tolerance to drought and UV radiation [2], and contributing to resistance against pathogens and pests [3].

Plant cuticular wax is mostly composed of very-long-chain fatty acids (VLCFAs), which typically include 20 to 40 carbon atoms. These VLCFAs serve as precursors for the formation of a variety wax components, including alkanes, aldehydes, primary and secondary alcohols, ketones, and esters [1, 4]. In addition to their role in cuticular wax, VLCFAs are important components of several lipid classes, such as phospholipids, sphingolipids, glycerophospholipids, triacylglycerols, and wax esters [5].

The cuticular synthetic process in plants consists of three steps: (1) *de novo* synthesis of C16 or C18 fatty acids in the plastid stroma; and (2) extension of these fatty acids in the endoplasmic reticulum (ER) to produce VLCFAs. These VLCFAs are produced by the ECERIFERUM2 (CER2) and fatty acid elongase (FAE) complex proteins [6]. The FAE complex includes β-ketoacyl-CoA synthase (KCS), β-ketoacyl-CoA reductase (KCR), 3-hydroxyacyl-CoA dehydratase (HCD), and enoyl-CoA reductase (ECR). These enzymes catalyze the condensation, reduction, dehydration, and reduction processes necessary for the synthesis of very-long-chain acyl-CoA [5, 7–9]. (3) VLCFAs are converted into main wax components such as aldehydes, alcohols, alkanes, ketones, and esters [4, 10].

KCS catalyzes the condensation step in the biosynthesis of VLCFAs, serves as the rate-limiting enzyme in the fatty acid elongation process, and has a defined substrate specificity [11, 12]. The first known *KCS* gene, *AtKCS18/FAE1*, was discovered in *Arabidopsis* and contributes to the catalysis of C20 to C26 VLCFAs in seeds [13]. Based on amino acid sequence homology, at least 21 potential *KCS* genes were identified in *Arabidopsis* and classified into four groups: FAE1, KCS1, FDH, and CER6 [14]. Furthermore, *Arabidopsis* KCS proteins are classified into eight subgroups based on the number and structural characteristics [15]. The *KCS* gene family has been discovered in many plant species, including 16 in *Citrus sinensis* [16], 28 in *Malus domestica* [17], 30 in *Arachis hypogea* [18], 58 in *Brassica napus* [19], and 58 in *Gossypium hirsutum* [20]. KCS proteins contain two conserved domains: an FAE1/type III polyketide synthase-like protein domain (FAE1_CUT1_RppA) and a 3-oxoacyl-[acyl-carrier protein (ACP)] synthase III C domain [18, 21]. The FAE1_CUT1_RppA domain has a substrate binding motif that is related with the substrate specificity of the KCS enzyme [22].

Several investigations have been conducted to investigate the function of *KCS* genes in plant lipid biosynthesis pathways. For example, the *Arabidopsis KCS5*/*CER60* gene has been linked to the synthesis of VLCFAs with chain lengths ranging from C26 to C30 [23]. Five *AtKCS* genes (*KCS1*, *KCS2*, *KCS6*/*CUT1*/*CER6*, *KCS9* and *KCS20*) are principally important for the production of cuticular wax and suberin precursors [24]. *AtKCS1* catalyzes VLCFA synthesis from C20 to C26, while *AtKCS9* participates in VLCFA elongation from C22 to C24 [25]. *AtKCS2/DAISY* and *AtKCS20* are functionally redundant in the elongation of VLCFAs from C22 to C24, which is necessary for the formation of cuticular waxes and root suberin [9]. The main function of *AtKCS6/AtCUT1/AtCER6* is to produce VLCFA above C28 for pollen coat lipids and cuticular waxes [26, 27]. Tetracosanoic acid, a precursor of phospholipids, sphingolipids, suberin, and cuticular waxes, is synthesized by *AtKCS9* [8]. In *Arabidopsis*, *AtKCS16* has been has been connected to the production of wax and the development of leaf trichomes [28]. *AtKCS7*, *AtKCS15*, and *AtKCS21* are all implicated in pollen coat lipid accumulation [29]. Additionally, *KCS* is involved in the synthesis of cuticular wax in *Citrinae* [16], rice (*Oryza sativa*) leaves [11], and oranges (*Citrus sinensis*) [30]. *HaKCS1* and *HaKCS2*, which catalyze the elongation of fatty acids from C18 to C24, are expressed in developing seeds of the sunflower during the oil accumulation phase [31].

The function of KCS in adapting to a range of environmental difficulties, including as drought, salt and cold stress, and biotic stress, has also been studied in the past. For example, *Arabidopsis kcs1* mutants exhibited reduced tolerance to low humidity settings during the early phases of development [32]. In groundnuts (*Arachis hypogaea*), overexpression of *AhKCS1* reduced water loss and membrane damage during drought stress by enhancing cuticular wax production in leaves [33]. Ectopic overexpression of *MdKCS2* from apple (*Malus domestica*) improved drought resistance in transgenic *Arabidopsis* by increasing wax accumulation [34]. Similarly, ectopic expression of *C. sinensis CsKCS6* in *Arabidopsis* improved drought tolerance [35]. Ectopic overexpression of grape (*Vitis vinifera*) *VvKCS* in *Arabidopsis* improved salt tolerance throughout the germination and seedling stages [36]. In cotton (*Gossypium hirsutum*), *GhKCS13* modulates the cold response by altering the lipids and oxylipin production [37]. Overexpression of *HvKCS1* in barley (*Hordeum vulgare*) increased leaf waxes and resistance to the barley powdery mildew fungus [38].

Sunflower is the world’s fourth most important oilseed crop. In China, it is mostly grown in dry and semi-arid northern regions [39]. Sunflower plants are frequently subjected to a number of abiotic and biotic stresses during their lives. Drought is one of the most major constraints to their growth and productivity [40–42]. Therefore, identifying stress tolerance genes in sunflower is crucial for understanding the mechanisms underlying drought stress response. Although previous research has demonstrated the importance of cuticular waxes in plant stress resistance, the role of wax-related genes in regulating wax synthesis in sunflower is not well understood. In this study, *HaKCS* genes were discovered in the sunflower genome database. Their expression patterns in diverse tissues, as well as under drought and ABA, were studied using quantitative real-time PCR (qRT-PCR). Using an *Arabidopsis* mutant with heterologous expression, the function of *HaKCS1-1* in cuticular waxes synthesis and drought tolerance was investigated. These findings provide insight into the molecular mechanisms underlying cuticular wax and VLCFA formation, as well as the drought stress response in sunflower.

## Materials and methods

### Identification of KCS gene family members in sunflower

To identify the *KCS* gene family in the sunflower genome, the genome file and protein sequences were downloaded from the Ensembl Plants (http://plants.ensembl.org/index.html). The *Arabidopsis* KCS protein sequences (*AtKCS*) [15] were obtained from TAIR (https://www.Arabidopsis.org/). The conserved KCS domain profiles (PF08541, 3-oxoacyl-[acyl-carrier-protein (ACP)] synthase III C-terminal; and PF08392, FAE1/type III polyketide synthase-like protein) were obtained from the Pfam protein family database (http://www.pfam.org/). The KCS protein sequences from sunflower genome data were obtained using local blastp and hidden Markov models. The sequence data acquired using the two approaches were combined, and redundant sequences or sequences lacking the two domains of KCS were removed. Finally, members of the sunflower *KCS* gene family were designated based on their homology to *Arabidopsis KCS* gene family members. The isoelectric point (pI) and molecular weight were estimated using ExPASy (https://www.expasy.org/). The transmembrane helices (TMHs) of HaKCSs were predicted using the TMHMM tool on the DTU Health Tech (https://services.healthtech.dtu.dk/).

### Phylogenetic tree and gene structure analysis

Multiple sequence alignments of KCS proteins from sunflower, *Arabidopsis*, lettuce (*Lactuca sativa*) and maize (*Zea mays*) were conducted using Clustal X software. A phylogenetic tree was created in MEGA 7.0 using the Neighbor-Joining method and 1000 bootstrap replications, and then displayed with the iTOL online tool (https://itol.embl.de/). GSDS 2.0 (http://gsds.gao-lab.org/) was used to construct exon–intron structures from genomic sequences [43].

### Conserved motif and cis-element analysis

The MEME tool (http://alternate.meme-suite.org/tools/meme/) was used to identify the *HaKCS* gene family conserved motifs [44]. PlantCARE (http://bioinformatics.psb.ugent.be/webtools/plantcare/html/) was used to identify *cis*-regulatory elements in the *HaKCS* genes’ promoter region (2000 upstream of the initiation codon). The functional distribution of the identified elements was visualized with TBtools [43].

### Chromosome distribution, synteny analysis and gene duplication

Circos software was used to analyze the synteny analysis of *KCS* homologous genes in *Arabidopsis*, sunflower and safflower (*Carthamus tinctorius* L.) [45]. Safflower genomic data was obtained from https://safflower.scuec.edu.cn/. The chromosomal distribution of *HaKCSs* was determined using sunflower annotation and visualized with MapGene2Chromosome V2 (http://mg2c.iask.in/mg2c_v2.0/). Multiple sequence alignment was used to identify gene duplication events, with a screening requirement of more than 70% similarity. The Simple Ka/Ks Calculator from the TBtools software package was used to calculate the Ka (non-synonymous substitution rate) and Ks (synonymous substitution rate) values. The Ka/Ks ratio was used to evaluate selection pressures on a gene, with results interpreted as neutral evolution (Ka/Ks = 1), purifying selection (Ka/Ks < 1), or positive selection (Ka/Ks > 1) [43].

### Plant materials and growth conditions

The expression of *HaKCS* genes was examined using the sunflower cultivar T303, a popular oil-sunflower cultivar in northwestern China. Stem, leaf, and cotyledon samples were collected from 4-week-old sunflower seedlings in an experimental field at Shihezi University in Xinjiang, China. To investigate the expression profile of *HaKCS* genes during seed development, sunflower seed embryos were collected at 10, 24, and 38 days after flowering (DAF), which correspond to the early, mid, and late stages of lipid accumulation. All samples were immediately frozen in liquid nitrogen and stored at -80 °C.

The *Arabidopsis* T-DNA insertion mutant *kcs1* (SALK_036681c, Columbia-0 ecotype) was obtained from the Nottingham *Arabidopsis* Stock Centre (NASC). The T-DNA insertion site is located 500 base pairs upstream of the transcription initiation site in *AtKCS1*’s promoter region (AT1G01120.1). *Arabidopsis* plants were grown in soil in a growth chamber at 22–24 °C (16 h light/8 h dark, 150 µmol photons m^− 2^ s^− 1^). Wild-type (WT) *Arabidopsis* (Col-0) plants was served as the control.

### Subcellular localization analysis

To estimate the subcellular localization of HaKCSs, their amino acid sequence file was submitted to the DeepLoc-2.1 website (https://services.healthtech.dtu.dk/services/DeepLoc-2.1/). TBtools was then used to visualize [46].

The location of *HaKCS1-1* was established via transient expression in *Arabidopsis* protoplasts. The full-length open reading frame (ORF) sequence of *HaKCS1-1*, which lacks a stop codon, was amplified using gene-specific primers P1 (Table S1). The PCR products were then inserted into the expression vector pAN580*-eGFP* to produce the fusion expression vector pAN580*-HaKCS1-1-eGFP*. This vector was then combined with an endoplasmic reticulum (ER) marker plasmid carrying *mCherry-HDEL-eRFP* and co-transformed into *Arabidopsis* protoplasts according to the protocol [47]. The empty vector *pAN580-eGFP* served as the control. GFP and mCherry signals were detected with a fluorescent microscope (Nikon, Tokyo, Japan).

### PEG and ABA treatments

Sunflower seedlings that were four-week-old were used for stress treatments. Plants were grown in a growth chamber with a 16-h light/8-h dark cycle, 25 °C/23°C, and 55–60% relative humidity. Sunflower seedlings were treated with 15% PEG 6000 for 0, 1, 3, 6, 12 and 24 h to simulate drought stress [48]. To examine their sensitivity to ABA, sunflower seedlings were treated with 100 µmol/L ABA for 0, 1, 3, 6, 12, and 24 h (or sterile water for controls). For every treatment, three biological duplicates were conducted. The stem and leaf were collected, immediately frozen in the liquid nitrogen, and stored at -80 °C.

### RNA extraction and qRT‑PCR analysis

Total RNA was isolated using the FastPure Plant Total RNA Isolation Kit (Vazyme, Nanjing, China) according to the manufacturer’s instructions. The cDNA was synthesized using PrimeScript™ reverse transcriptase (TaKaRa, Dalian, China). qRT-PCR was performed on a Roche LightCycler 480 System with ChamQ SYBR qPCR Master Mix (Vazyme, Nanjing, China). The sunflower 18 S rRNA gene (GenBank: AF107577) was used as an internal control due to its high conservation, abundant expression, stability, and low susceptibility to RNA degradation in eukaryotes [49, 50]. The qRT-PCR data were evaluated with the 2^−∆∆CT^ technique [51]. The primers for qRT-PCR are listed in Table S1.

### Expression of *HaKCS1-1* gene in *Arabidopsis kcs1* mutant

The full-length coding sequence of the *HaKCS1-1* gene was inserted into the pCAMBIA2300 vector, which is driven by the *CaMV35S* promoter, using gene-specific primers (K1) with *Bam*H I and *Sal* I sites (Table S1). The recombinant plasmid was introduced into *Agrobacterium tumefaciens* GV3101, which was then transformed into the *Arabidopsis kcs1* mutant via the floral dip method [52]. The *Arabidopsis* wild type (Col-0) was used as a control. Transgenic T1 plants were selected on MS medium with kanamycin (50 mg/L) and confirmed by PCR with primers K1. The expression of *HaKCS1-1* in the positive lines was verified with semi-quantitative RT-PCR. Homozygous T_3_ transgenic lines were selected for further study.

### Scanning Electron Microscopy (SEM)

To study the morphology of epicuticular wax crystals on *Arabidopsis* leaves and stems, rosette leaves and stems were collected from 6-week-old WT, *kcs1* mutant, and *HaKCS1-1* transgenic plants, as previously described [53]. The epicuticular wax crystals were examined with a scanning electron microscope (JSM-6610lv, JEOL).

### TB staining and chlorophyll leaching assays

Toluidine blue (TB) staining was performed on 6-week-old healthy *Arabidopsis* leaves by immersing them in a 0.05% TB staining solution for 10 min, followed by 2–3 rinses with distilled water. For the chlorophyll leaching assay, the detached leaves were weighed and immersed in 80% ethanol. The chlorophyll leaching content was then measured according to the method of Zhang et al. (2019).

### Drought stress treatment and physiological indexes analysis

To investigate drought tolerance in transgenic *Arabidopsis*, drought stress was applied to 4-week-old WT, *kcs1* mutant and transgenic plants for 10 days. The plants were then rewatered for 3 days, and their survival rates were calculated. Water loss rates of *Arabidopsis* leaves were assessed using the methods described in Dai et al. [54].

### Extraction of cuticular wax and GC-MS analysis

Approximately 100 mg of stems from 6-week-old wild type, *kcs1* mutant and *HaKCS1-1* transgenic *Arabidopsis* plants were collected, and total waxes were extracted by soaking the stems in chloroform for 30 s. After wax extraction, the stems were analyzed using ImageJ software (https://imagej.net/) [55]. To serve an internal standard, 10 µL of n-tetracosane (1 µg/µL) was added to each wax extract. After drying under nitrogen gas, the extracts were derivatized for 45 min at 70 °C by adding 20 µL of bis (N, N-trimethylsilyl)-trifluoroacetamide (BSTFA) and 20 µL of pyridine. Gas chromatography-mass spectrometry (GC–MS) was then used to identify the components of cuticular wax, including fatty acids, aldehydes, alcohols, alkanes, and esters [56]. The wax compounds were identified based on their electron ionisation mass spectrometry spectra, and their amounts were calculated relative to the internal standard. Three biological replicates were performed for each treatment.

### Statistical analysis

The data were analyzed using SPSS 26.0 software, which included the Student’s t-test and one-way ANOVA. Statistical significance was determined at *P* < 0.01 and *P* < 0.05 for t-test, and at *P* < 0.05 for Duncan’s multiple range tests. All data are reported as the mean ± SD (*x*±*s*) of three biological replicates and were plotted using GraphPad Prism 8.0.2.

## Results

### Identification of the *HaKCS* genes in sunflower

A total of 26 HaKCS proteins were found from the sunflower genome using the hidden Markov model (HMM) profile of the *KCS* gene family and confirmed with local Blastp (Table S2). The whole FAE1_CUT1_RppA and ACP_syn_III_C domains are present in all HaKCS proteins.

The HaKCS protein family’s molecular weights range from 36.37 to 63.58 kDa, and its amino acid sequences range from 327 aa (HaKCS4-1) to 574 aa (HaKCS4-2). The length difference between the longest (HaKCS4-2) and shortest (HaKCS4-1) members is 247 aa. This substantial variation in sequence length, particularly within functional domains, suggests that different HaKCS proteins may have diversified substrate specificities or tissue-specific roles. Their predicted pI values ranged from 8.09 to 9.56, indicating potential interactions with negatively charged molecules such as nucleic acids or acidic protein partners.

### Phylogenetic relationships and classification of KCS proteins

**Fig. 1:**
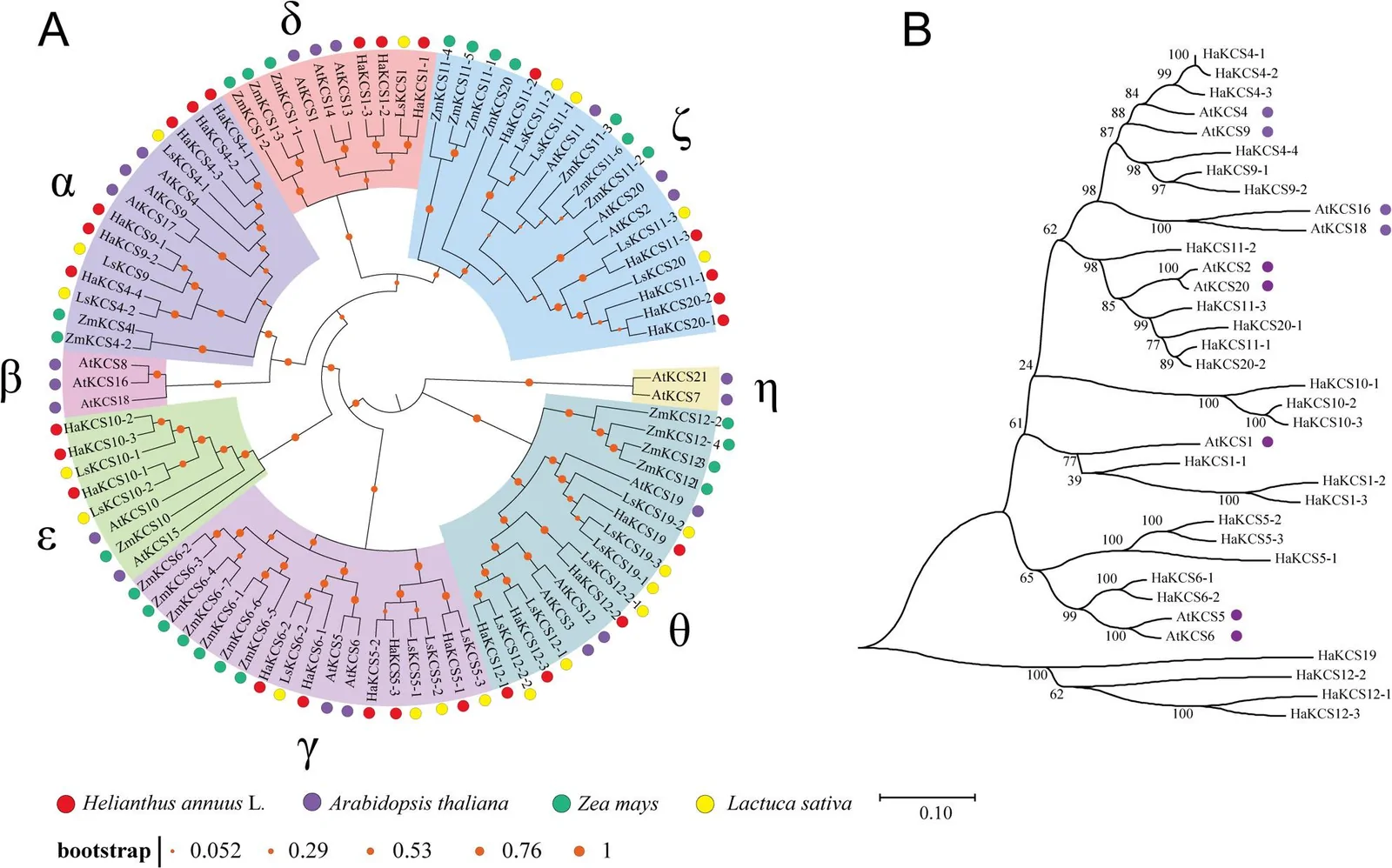
Phylogenetic study of the KCS family. **A** A phylogenetic tree was constructed using KCS protein sequences from four species: *Helianthus annuus* L. (Ha, red), *Arabidopsis thaliana* (At, purple), *Zea mays* (Zm, green), and *Lactuca sativa* (Ls, yellow). The phylogenetic tree was divided into eight subgroups, with each clade represented by a different hue. **B** A phylogenetic tree was built by combining sunflower HaKCSs and nine *A. thaliana* KCS members of well-known functions

To study potential functional divergence within the KCS protein family, a phylogenetic tree was built using KCS homologs from four species: *Helianthus annuus* (Ha), *Arabidopsis thaliana* (At), *Lactuca sativa* (Ls), and *Zea mays* (Zm) (Fig. 1; Table S3). Previous research has classified the 21 *Arabidopsis KCS* genes into four major subfamilies based on sequence homology: *KCS1*-like, *FAE1*-like, *FDH*-like and *CER6*-like [14, 57]. The subfamilies are further categorized as eight subgroups: α, β, γ, δ, ζ, ε, η and θ [18]. The β and η subgroups did not contain any members of the sunflower KCS family. The remaining subgroups contain the following genes: α (6), γ (5), δ (3), ζ (5), ε (3) and θ (4). HaKCS proteins are most closely related to those discovered in *L. sativa*, another member of the Asteraceae family. Furthermore, unlike the monocotyledonous *Z. mays*, the KCS proteins in sunflower, *Arabidopsis*, and *L. sativa* are more closely related.

To investigate the potential functional divergence among sunflower HaKCS family members, a phylogenetic tree was built using nine functionally reported AtKCS proteins involved in cuticular wax biosynthesis, as well as 26 HaKCS proteins (see Fig. 1B). The findings revealed distinct patterns of clustering among AtKCSs and HaKCSs. HaKCS1-1, HaKCS1-2 and HaKCS1-3 clustered inside the same branch as AtKCS1, while HaKCS4-1, HaKCS4-2 and HaKCS4-3 clustered with AtKCS4. HaKCS6-1 and HaKCS6-2 come from the same branch as AtKCS6 and AtKCS5, respectively. Based on the evolutionary relationship, we anticipate that HaKCS members clustered from the same branches may share biological activities with their *Arabidopsis* homologues. This information is useful for future functional analyses of HaKCS proteins.

### Gene structure and conserved motif analysis

**Fig. 2:**
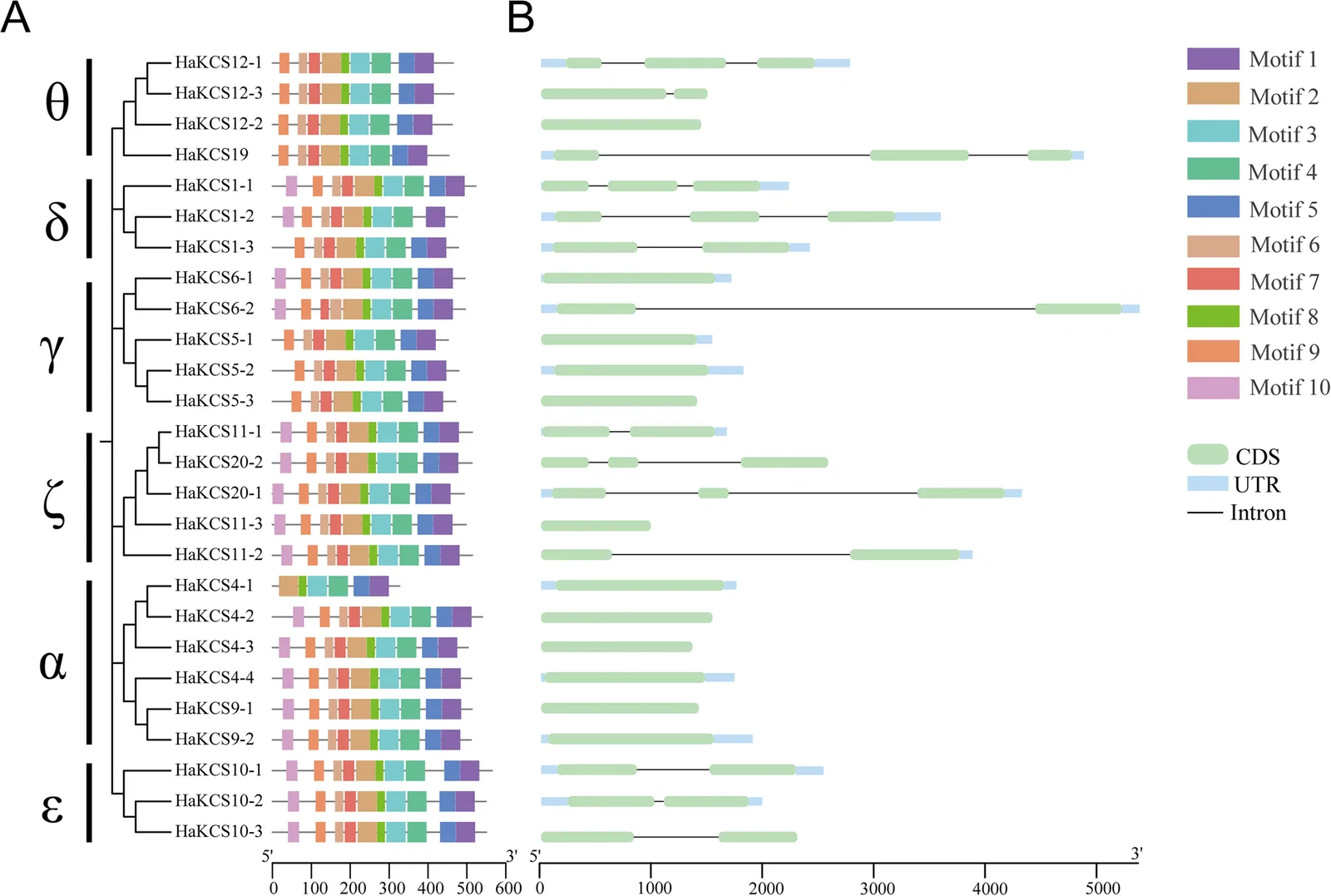
Conserved motifs and gene structures of *HaKCS* genes in sunflower. **A** The distribution of ten conserved motifs among the 26 HaKCS proteins. The motifs are represented as colored boxes, each with a distinct color and number. **B** The exon-intron structures of *HaKCS* genes. Exons are depicted by green boxes, and introns by black lines

To assess structural variation and evolutionary limits among HaKCS proteins, the conserved motif composition and exon–intron arrangement were comprehensively examined. MEME analysis revealed ten distinct common motifs among the HaKCS proteins (Fig. 2A; Table S4). Fifteen members (57%) have all ten motifs, seven (27%) have nine motifs, one (12%) has eight, and three (4%) have seven motifs. Sequences with high homology generally share conserved motifs. The three members of the ε subgroup (HaKCS10-1, HaKCS10-2, and HaKCS10-3) have ten conserved motifs and identical gene structures. Except for HaKCS4-1, all five HaKCS proteins from the α subgroup contain ten conserved motifs.

Four highly conserved regions have been identified in the HaKCS protein domain: GMGCSA, PTPSLSAM, FGNTSSSS and GSGFKCNSAVW. Motif 2 is made up of GMGCSA, the KCS enzyme’s catalytic domain, which includes the cysteine residue that acts as the catalytic site. Motif 4 contains the ACP_syn_III_C domain. FAE1_CUT1_RppA (PF08392) and ACP_syn_III_C (PF08541) domains are present in both *Arabidopsis* and sunflower KCS proteins, indicating that these are crucial functional regions for KCS proteins.

To assess the evolutionary conservation of the *HaKCS* genes, their gene structures were analyzed (Fig. 2B). The *HaKCS* gene family contains one to three exons. Twelve genes include one exon, eight have two, and six have three. The majority of *HaKCS* genes in the same subgroup share identical or highly similar gene structures. For example, all five *KCS* genes in subgroup ε from sunflower have three exons and two introns. Overall, the *HaKCS* genes in each subgroup have similar conserved motifs and gene structures.

### Cis-element composition of *HaKCS* genes

**Fig. 3:**
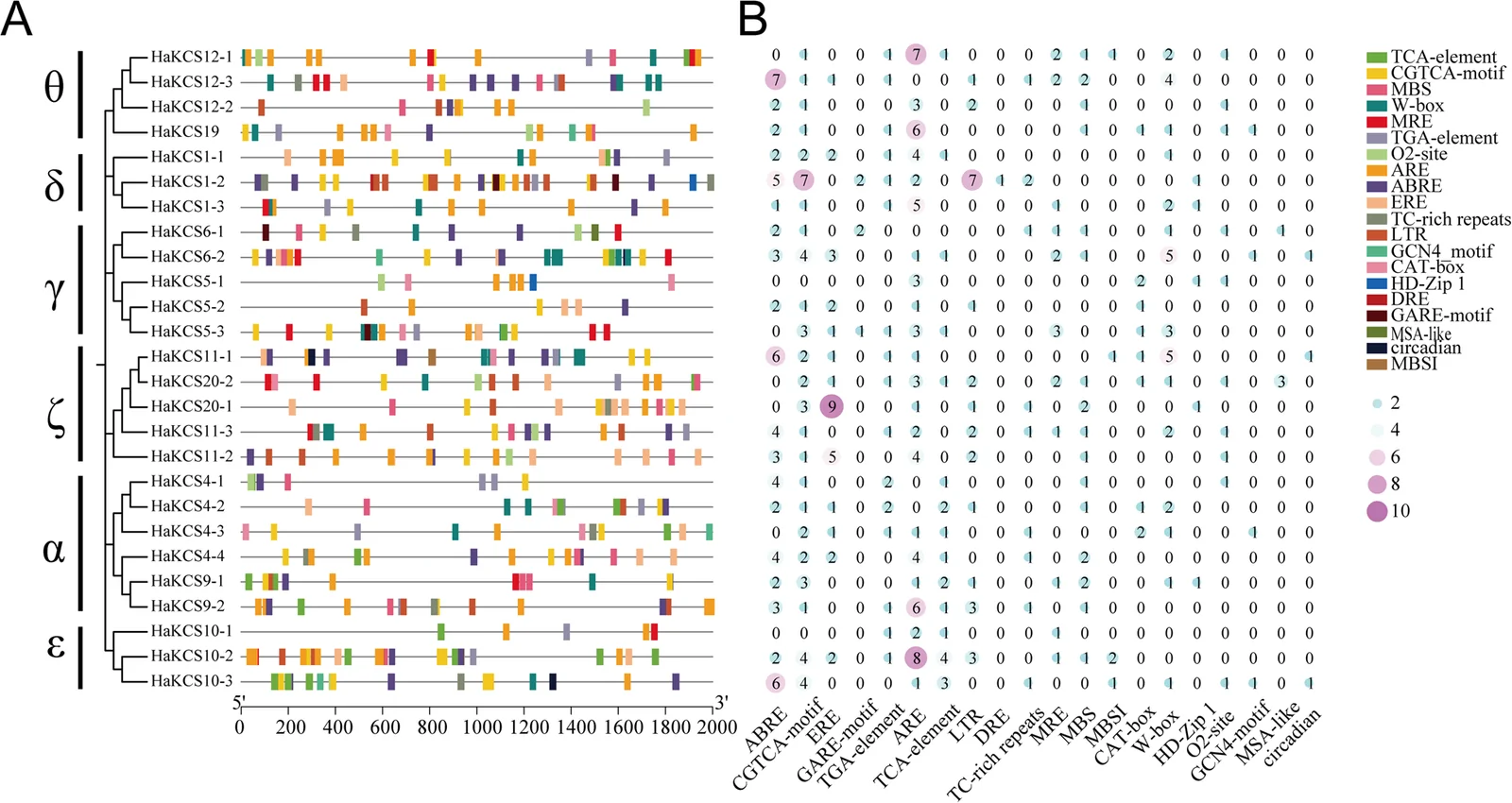
*Cis*-acting regulatory elements in *HaKCS* gene promoters. **A** Predicted cis-element distribution in *HaKCS* promoters. Colored boxes denote distinct element kinds. The gene order corresponds to the evolutionary tree shown on the left. **B** A heatmap depicting the distribution of each cis-element type among *HaKCS* promoters

To investigate possible transcriptional regulation mechanisms, the 2,000 bp areas upstream of each *HaKCS* gene were examined for *cis*-acting elements. The *HaKCS* promoters include twenty different types of *cis*-regulatory elements. These components were divided into three categories based on their anticipated functions: plant growth and development, phytohormone responsiveness, and abiotic and biotic stress (Fig. 3A, B).

The majority of these *cis*-regulatory elements were associated with stress reactions, both biotic and abiotic. These included anaerobic induction, dehydration, low temperature, salt stress, drought, defense and pressure responses, accounting for 42% of the 169 characteristics. For example, the anaerobic response element (ARE), the low-temperature response element (LTR), the MYB binding site involved in light response (MRE), the MYB binding sites involved in drought induction (MBS), and the MYB binding site involved in flavonoid biosynthesis gene regulation (MBSI).

The second most common group, comprising 166 components (41%), was related to phytohormone responsiveness. This category included elements that respond to ABA, ethylene, gibberellin, methyl jasmonate, auxin, salicylic acid and flavonoids. In contrast, *cis*-regulatory elements involved in plant growth and development were the least common, accounting for only 17% of the total. These include the CAT-box (related with meristem expression), the GCN4_motif (associated with endosperm expression), the HD-Zip motif (associated with palisade mesophyll cell differentiation) and the MSA-like motif (involved in cell cycle control). The MSA-like motif was discovered in *HaKCS6-1* and *HaKCS20-2*, whereas the GCN4_motif was found in *HaKCS4-3*, *HaKCS6-2*, *HaKCS10-3*, *HaKCS11-2* and *HaKCS19*.

The three *cis*-acting element types, ARE, ABRE (ABA response element), and the CGTCA motif (methyl jasmonate response element), were broadly distributed. These were discovered in the 22, 19 and 24 *HaKCS* promoters, respectively. In contrast, the GARE motif (gibberellin-responsive element), was discovered only in *HaKCS1-2*, *HaKCS4-1*, *HaKCS5-3* and *HaKCS6-1*. Only *HaKCS1-2* contains the DRE element, which is important in responses to dehydration, low temperatures, and salt stress. This circadian-related element was only detected in *HaKCS10-3*, *HaKCS11-1*, and *HaKCS6-2*. Twelve *HaKCS* promoters had the LTR (low-temperature response element), while fourteen contained the MBS (MYB binding site, which is essential for drought induction).

### Chromosomal localization and duplication events of *HaKCS* genes

**Fig. 4:**
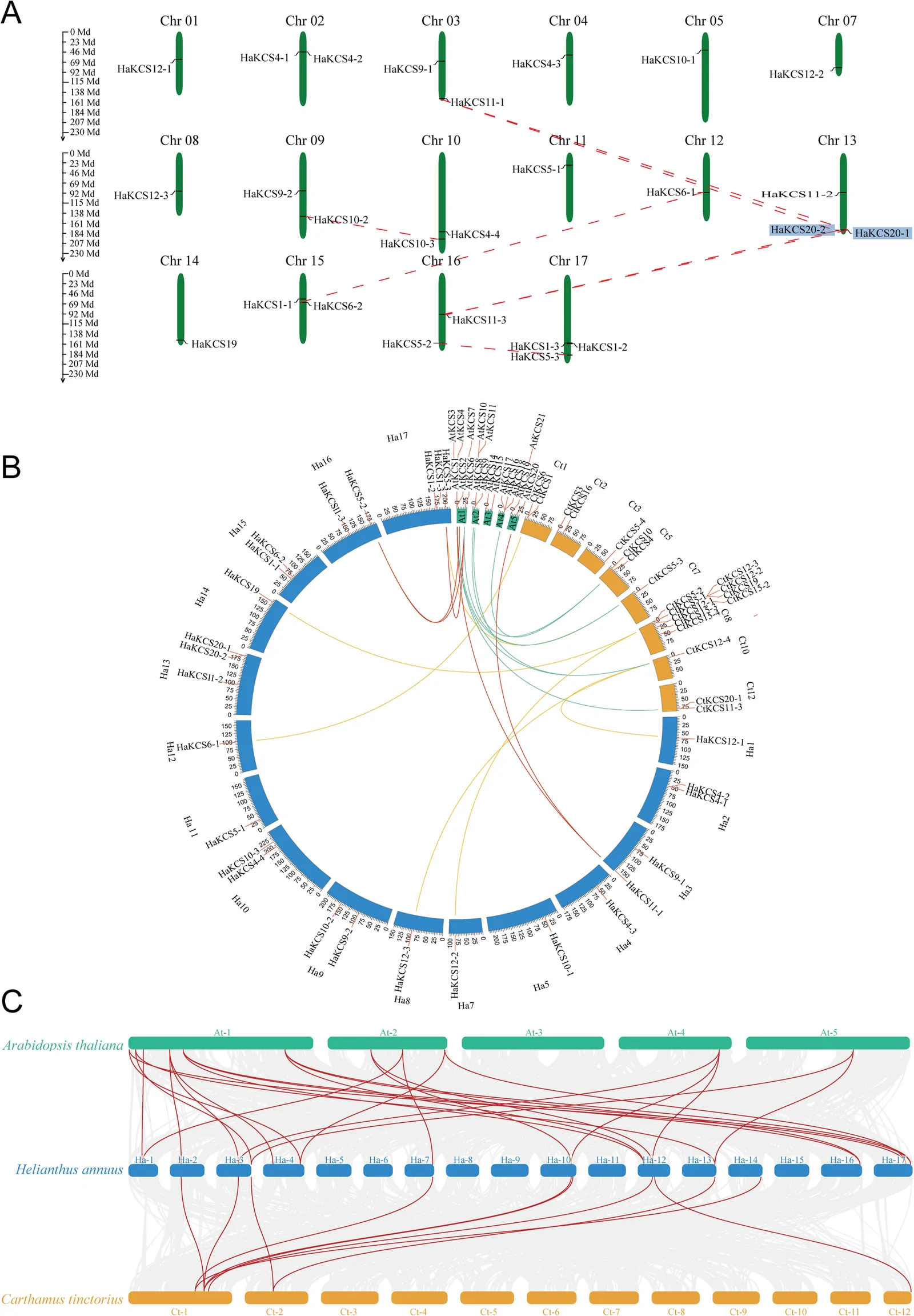
Chromosomal location and synteny analysis of *HaKCS* genes. **A** locations of the *HaKCS* genes on the 16 sunflower chromosomes. Blue boxes represent tandemly repeated genes, whereas red dashed lines represent segmentally repeated genes. The size on the left shows the sunflower’s chromosomal length (Mb). **B**,** C** Synteny study of *KCS* genes in *Helianthus annuus* L. (Ha), *Arabidopsis thaliana* (At) and *Carthamus tinctorius* (Ct)

According to the sunflower genome sequence, 26 *HaKCS* genes were distributed among the 16 sunflower chromosomes, with the exception of chromosome 6 (Fig. 4A). The majority of *HaKCS* genes were located in the inner regions of chromosomes, with a small number located at the telomeric ends. The *HaKCS* genes were distributed relatively evenly throughout the sunflower genome. One gene was detected on chromosomes 1, 4, 5, 7, 8, 11, 12, and 14, two on chromosomes 2, 3, 9, 10, 15, and 16, and three on chromosomes 13 and 17.

Gene duplication study revealed that the sunflower genome has a single tandem duplication event, HaKCS20-1/HaKCS20-2 on chromosome 13. Seven pairs of segmental duplication events were also detected, implying that these events were crucial in the expansion of the *HaKCS* genes throughout evolution. To further understand the evolutionary limitations that affect the *HaKCS* genes, the Ka/Ks ratios for *HaKCS* gene pairs were calculated **(**Table S5). The results revealed that the Ka/Ks ratios of all duplicated gene pairs were less than one, indicating that the *HaKCS* genes are exceptionally well-preserved and have most likely undergone strong purifying selection over time. We created a syntenic map of safflower, sunflower, and *Arabidopsis* in order to better understand the evolution of the *KCS* genes (Fig. 4B, C). The results showed that nine *Arabidopsis AtKCS* genes and eight safflower *CtKCS* genes in safflower are orthologs of sunflower *HaKCS* genes.

### Tissue-specific, drought, and ABA-induced *HaKCSs* expression patterns

**Fig. 5:**
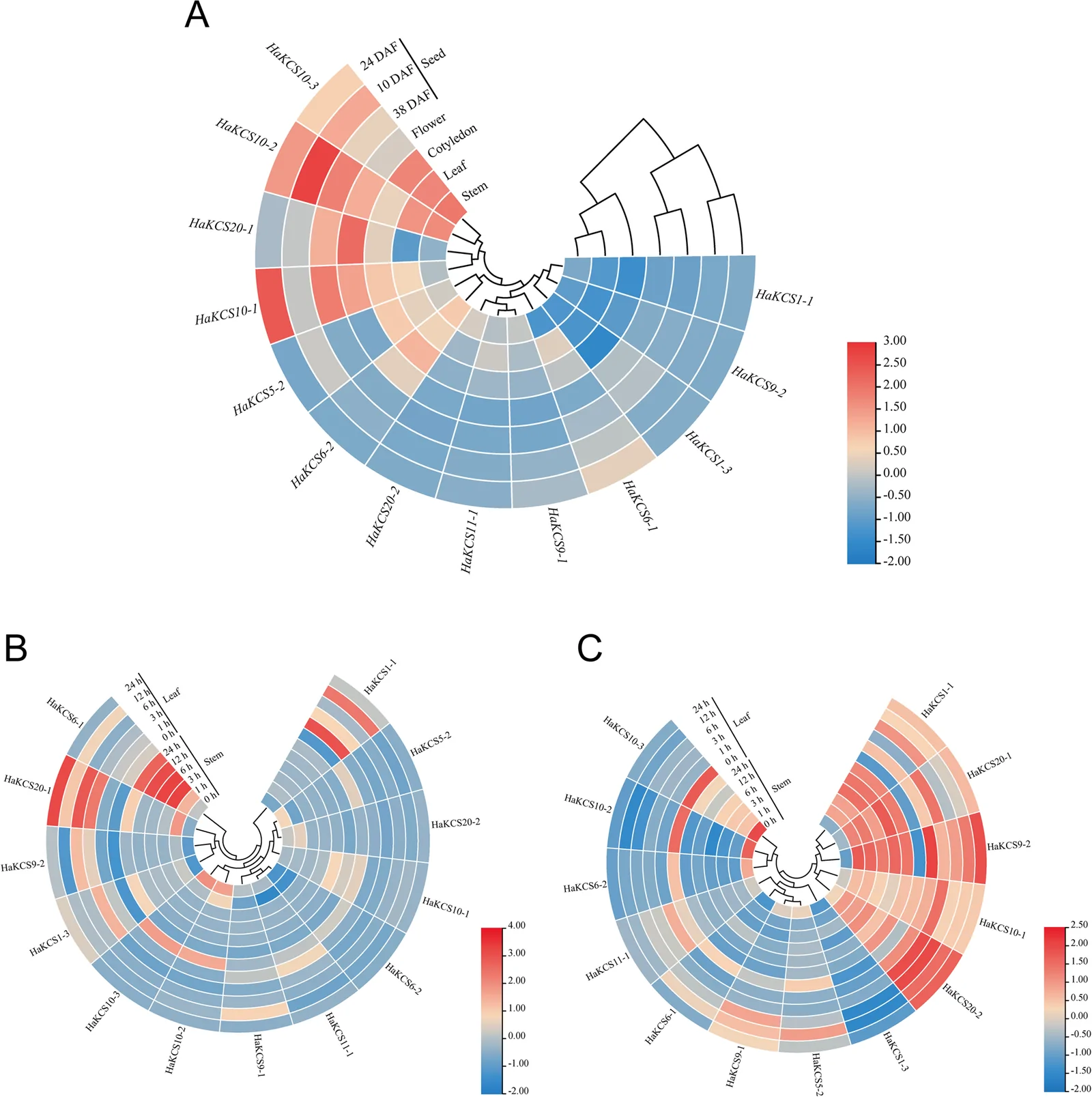
Expression profiles of *HaKCS* genes in different sunflower tissues following drought and ABA treatments. **A** Heatmap showing the expression levels of *HaKCS* genes in sunflower stems, leaves, cotyledons, flowers, and developing seeds at 10, 24, and 38 Days After Flowering (DFA). **B** A heatmap showing 13 *HaKCS* genes expression levels following PEG 6000 treatment. Sunflower seedlings were treated with 15% PEG 6000 (w/v) for 0, 1, 3, 6, 12 and 24 h. **C** Heatmap of 13 *HaKCS* genes expression levels following ABA treatment. Sunflower seedlings were treated with 100 µmol/L ABA for 0, 1, 3, 6, 12 and 24 h, respectively. Each treatment was tested in three separate replicates. Red and blue represent the *HaKCS* genes that are highly and weakly expressed after treatment, respectively. Based on the qRT-PCR results, a visual heatmap was produced using the R programming language

To investigate the potential biological roles of *HaKCSs*, 13 *HaKCS* genes from six subgroups were chosen for qRT-PCR in five different sunflower tissue types: stems, leaves, cotyledons, flowers, and developing seeds (10, 24, and 38 DAF) (Fig. 5A). The heatmap showed *HaKCS* gene expression levels varied throughout sunflower tissues. Interestingly, the stem, leaf, and cotyledon exhibit the highest levels of *HaKCS* gene expression. The majority of *HaKCS* genes were low in expression in flowers and seeds, with the exception of *HaKCS10-1*, which was highly expressed in developing seeds at 24 DAF. These tissue-specific expression patterns suggest that *HaKCS* genes may primarily function in vegetative organs to regulate cuticular wax biosynthesis and protect against environmental stresses, but some members, such as *HaKCS10-1*, may play a specialized role in seed lipid elongation and storage.

The transcript levels of *HaKCS* genes in sunflower stems and leaves under drought stress (15% PEG 6000) were further investigated using qRT-PCR (Fig. 5B). The results revealed that the majority of *HaKCS* genes had reduced expression levels in the leaves after 1 h of drought stress. Nonetheless, the stems showed increased amounts of most *HaKCS* genes, including *HaKCS1-1*, *HaKCS1-3*, *HaKCS6-1*, *HaKCS9-2*, *HaKCS20-1*, and *HaKCS20-2*. This stem-specific upregulation suggests that these *HaKCS* genes may contribute to the synthesis of cuticular waxes in stems, potentially enhancing stem-mediated protective barriers under drought conditions.

Nearly half of the *HaKCS* expression in stems was up-regulated after 100 µmol/L ABA treatment (Fig. 5C). For instance, the expression levels of *HaKCS1-1*, *HaKCS6-1*, and *HaKCS20-2* all peaked after 6 h of ABA. After 6 h of ABA, the expression levels of *HaKCS1-1*, *HaKCS9-1*, and *HaKCS20-2* peaked in leaves. *HaKCS9-2* and *HaKCS11-1* had the highest expression levels after 3 h of ABA. Meanwhile, after 1 and 3 h of ABA, the expression of *HaKCS5-2*, *HaKCS6-1*, and *HaKCS6-2* was down-regulated; but at 6 and 12 h, it was up-regulated. The results showed that the *HaKCS* genes were ABA-sensitive.

### Subcellular localization of *HaKCS* proteins

**Fig. 6:**
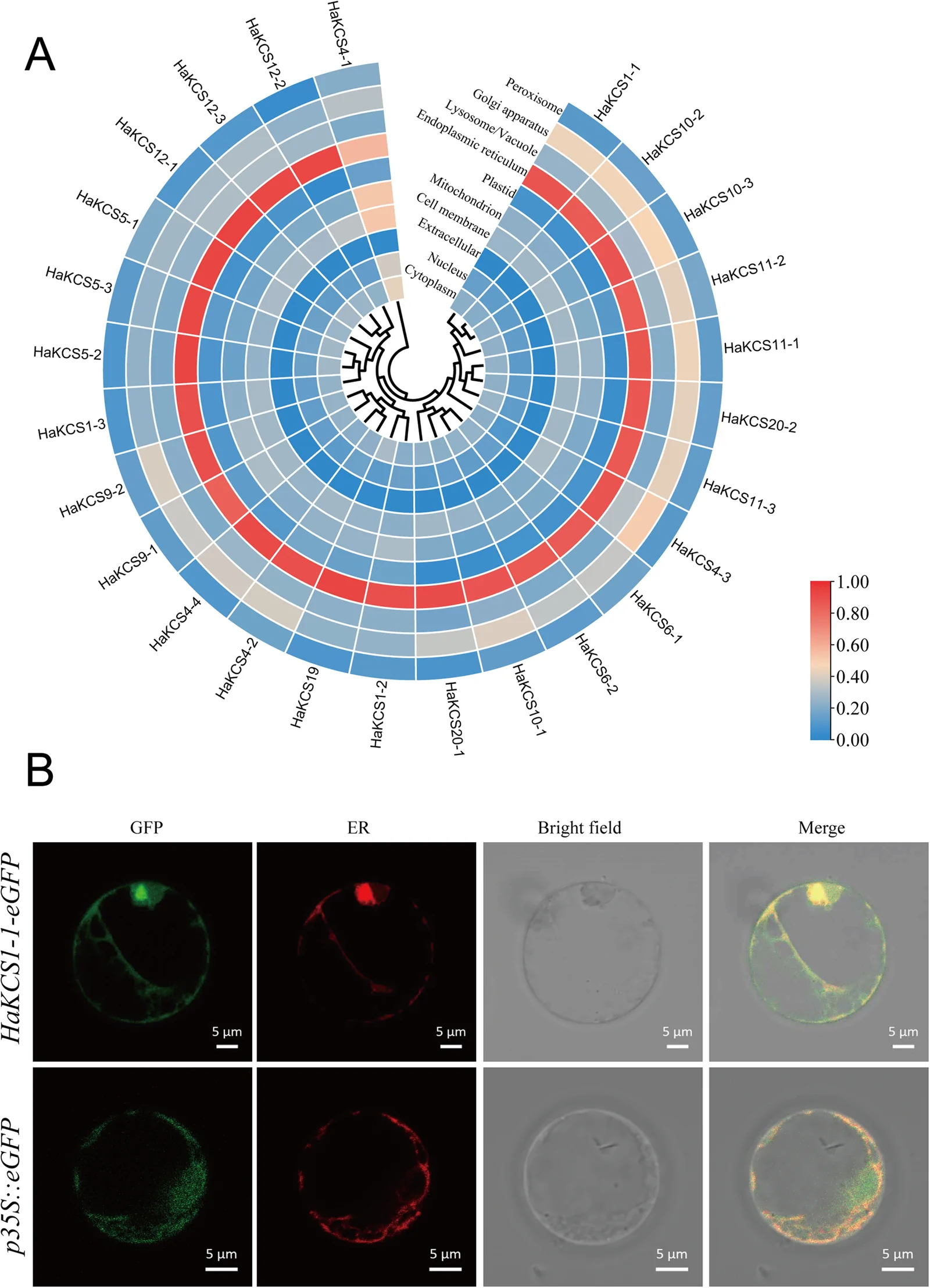
Subcellular localization of HaKCSs. **A** Subcellular Localization Prediction Heatmap of HaKCSs. **B** Subcellular localization of *HaKCS1-1* in the *Arabidopsis* protoplasts. *HaKCS1-1-eGFP* (green) coupled with Endoplasmic Reticulum (ER) marker (red). The p35s:: *eGFP* empty vector served as a control

To examine the biological roles of HaKCS enzymes, we predicted their subcellular localization for HaKCS family members (Fig. 6A). The results showed that all HaKCS proteins had the highest prediction scores for the endoplasmic reticulum (ER), implying that HaKCS family members are most likely localized to the ER in sunflower cells.

HaKCS1-1 shares 69.89% homology with *Arabidopsis* KCS1 and belongs to the δ-subclass. Because *AtKCS1* is strongly expressed during *Arabidopsis* growth and needed for cuticular wax deposition [24], we chose *HaKCS1-1*, the evolutionary closest homologue, for subcellular localization to further investigate the putative function of *HaKCSs*. We cloned the *HaKCS1-1* gene and created the *pAN580-HaKCS1-1-eGFP* recombinant vector, which contains green fluorescent protein (GFP). The *mCherry-HDEL-RFP* plasmid was then co-transformed into *Arabidopsis* protoplasts, allowing for transient expression. Confocal imaging revealed that the mCherry-HDEL and HaKCS1-1 proteins, which carry RFP and GFP respectively, were co-localized in the ER (Fig. 6B). This is consistent with results of the HaKCS1-1 subcellular localization prediction analysis (Fig. 6A**)**.

Overexpression of *HaKCS1-1 *changed the morphology and composition of the cuticular waxes in the *Arabidopsis kcs1 *mutant.

The cDNA of *HaKCS1-1* was cloned and introduced into *Arabidopsis kcs1* mutant under the control of the CaMV 35 S promoter. PCR was performed with *HaKCS1-1* specific primers to detect positive transformants. The transcription levels of *HaKCS1-1* in transgenic lines of the T2 generation were investigated further using semi-quantitative RT-PCR (Fig.S1). These results showed that *HaKCS1-1* was highly expressed in the *Arabidopsis kcs1* mutant.

**Fig. 7:**
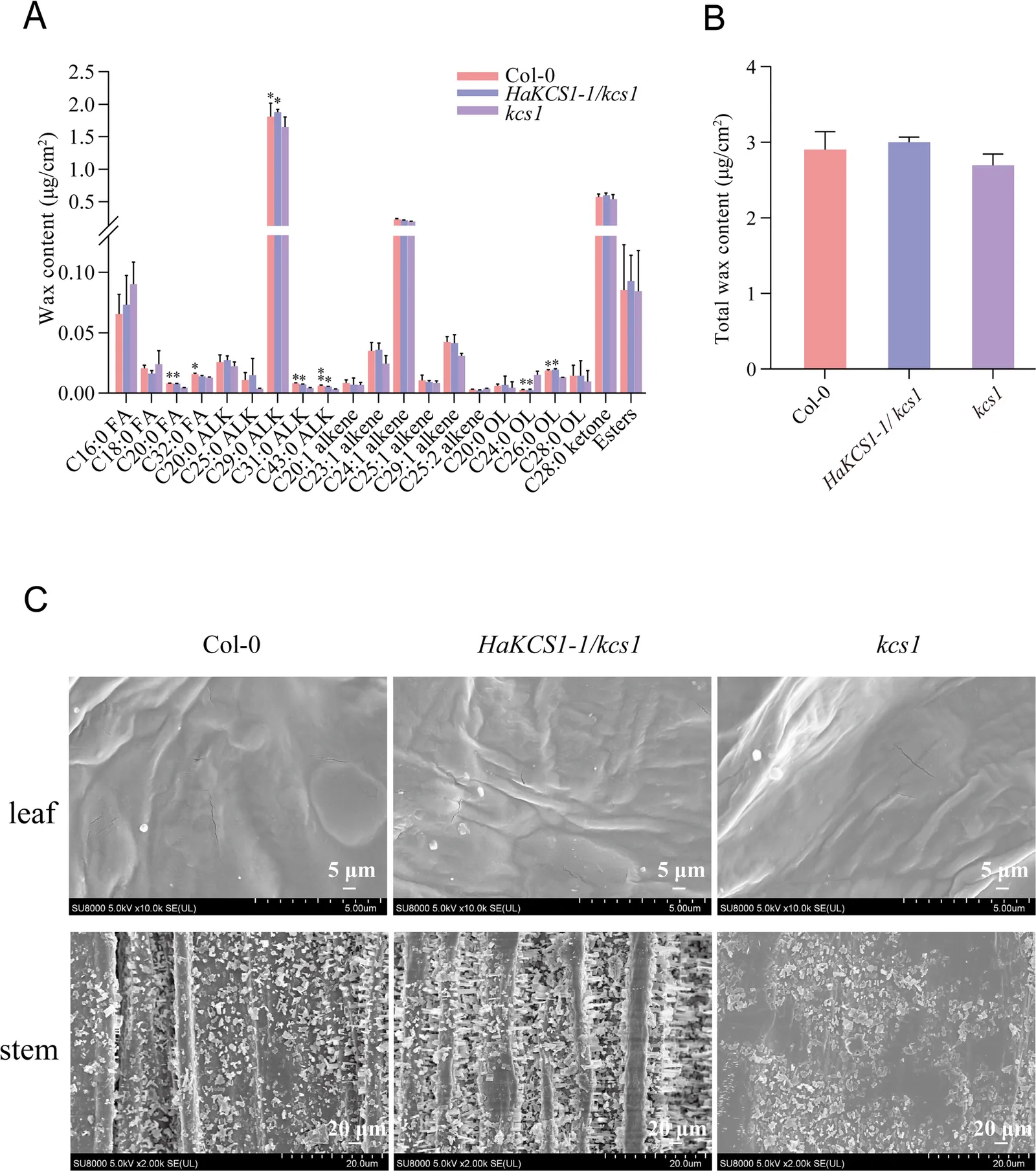
Analysis of cuticular wax in *Arabidopsis kcs1* mutants overexpressing *HaKCS1-1*. **A** Wax components in *Arabidopsis* stems: wild type (Col-0), *HaKCS1-1/kcs1* and *kcs1* mutant plants. **B** Total wax on the *Arabidopsis* stem. Asterisks represent statistically significant differences (*P < 0.05, **P < 0.01). Alcohols (OL); Alkanes (ALK); and Fatty Acids (FAs). **C** Scanning electron microscopy analysis of *Arabidopsis* leaf and stem cuticular waxes

To determine the effect of *HaKCS1-1* on wax composition, GC-MS analysis was performed on the wax content and composition on the stem surface of *Arabidopsis* wild-type (WT), *HaKCS1-1/kcs1* and *kcs1* mutant (Fig. 7A). The *kcs1* mutant has considerably lower C20:0 and C32:0 fatty acid contents than the WT. However, compared to the *kcs1* mutant, the *HaKCS1-1* transgenic plants produced considerably more of these fatty acids. For C29:0, C31:0, and C43:0, the *kcs1* mutant showed much lower alkane content than the WT. However, as compared to the *kcs1* mutant, the *HaKCS1-1* transgenic plants had considerably higher alkane levels. Furthermore, compared to the *kcs1* mutant, *HaKCS1-1* transgenic plants had a considerably higher C26:0 alcohol contents. The total wax load of *HaKCS1-1* transgenic plants was comparable to that of the WT, suggesting that it partially restores wax biosynthetic function in the *kcs1* mutant (Fig. 7B). These findings indicate that overexpressing *HaKCS1-1* in the *Arabidopsis kcs1* mutant can alter the composition of cuticular waxes, increase VLCFAs synthesis ability, and counteract the accumulation of shorter-chain fatty acids (C16, C18) caused by *KCS1* deficiency.

We also used scanning electron microscopy to analyze the wax morphology on the leaf and stem surfaces of Col-0, *HaKCS1-1*/*kcs1*, and *kcs1* plants. The results revealed that all three lines (WT, *HaKCS1-1*/*kcs1*, and the *kcs1* mutant) had similar wax structures on leaves (Fig. 7C). The *kcs1* mutant had a lower number and density of wax crystals than the WT, with less rod-shaped crystals on the stem surface and fewer epidermal crystals in the stems. However, the wax crystal structure in the stems of *HaKCS1-1*/*kcs1* transgenic *Arabidopsis* was similar to that of WT (Fig. 7C). This implies that the *Arabidopsis kcs1* mutant’s stem surface wax deficit was corrected by the *HaKCS1-1* gene.

### Overexpression of HaKCS1-1 enhanced drought tolerance in kcs1 mutants and reducing cuticular wax permeability

**Fig. 8:**
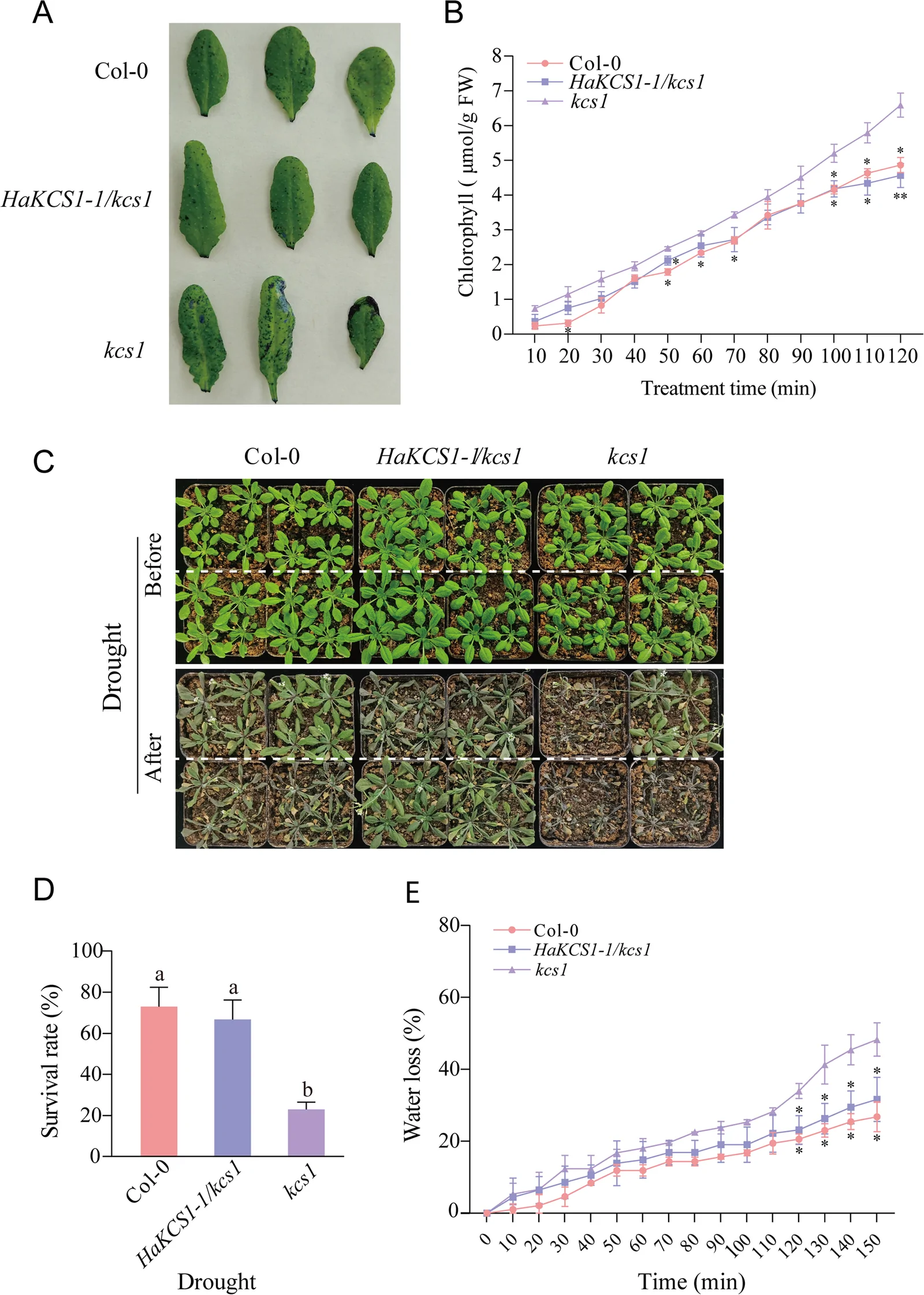
*HaKCS1-1* overexpression modifies cuticle permeability and improves drought tolerance in the *Arabidopsis kcs1* mutant. **A** TB staining. **B** The chlorophyll leaching content of *Arabidopsis* plants following alcohol treatment. **C** Phenotypes of wild-type (Col-0), the *HaKCS1-1* overexpression line and *kcs1* mutant under drought stress. Plants were cultivated under normal conditions for 4 weeks, then subjected to drought for 10 d, followed by three days of rewatering \ to allow recovery. **D** A statistical analysis of survival rates following three days of rewatering. **E** Water loss rates of detached rosette leaves of the *HaKCS1-1* overexpressing line, *kcs1* mutant and wild-type *Arabidopsis*. Data were presented as means ± SD from three independent replications. Asterisks indicate significant differences (*P < 0.05, **P < 0.01). Different letters indicate significant variations in values (*P* < 0.05)

Toluidine blue (TB) was used to stain the leaf epidermis of three different plant types to compare the permeability differences between WT, *HaKCS1-1*/*kcs1*, and *kcs1* plants. The rate of chlorophyll leakage was determined following treatment with 85% ethanol. The results showed that the *kcs1* mutant had more dots on the leaves and a higher TB staining. In contrast, the *HaKCS1-1*/*kcs1* transgenic strains had lower TB staining (Fig. 8A). After being treated with 85% ethanol, chlorophyll leaching rates of three different plant types were all increased. However, at all of the time point, *HaKCS1-1*/*kcs1* plants had consistently lower rates than *kcs1* mutants (Fig. 8B).

To determine whether *HaKCS1-1* contributes to the plant response to drought stress, *Arabidopsis* plants from three strains—WT (Col-0), *HaKCS1-1/kcs1*, and *kcs1*—were examined for phenotypic, survival rate, and water loss under drought treatment of *Arabidopsis* seedlings. The survival rates were assessed after three days of rehydration and ten days under drought stress. The findings revealed that *kcs1* mutants wilted significantly more than WT and *HaKCS1-1*/*kcs1* transgenic plants (Fig. 8C). After rehydration, *HaKCS1-1/kcs1* and Col-0 plants fared substantially better than *kcs1* mutants (Fig. 8D). Furthermore, at 120–140 min, detached rosette leaves in *HaKCS1-1*/*kcs1* and WT plants lost considerably less water than the *kcs1* mutant (Fig. 8E). Overall, heterologous expression of *HaKCS1-1* in *kcs1* mutants decreased drought sensitivity.

## Discussion

### Evolutionary conservation and expansion of the *HaKCS* gene family

This study found 26 *HaKCS* genes in the sunflower genome. All HaKCS proteins contain the FAE1_CUT1_RppA and ACP_syn_III_C domains, which are conserved in higher plant KCS proteins and essential for enzymatic action [20, 21, 58]. The FAE1_CUT1_RppA domain is particularly important as it harbors the catalytic residues and substrate-binding motifs [22], and it is the key catalytic region of KCS proteins.

Comparative genomic study between species can reveal important information about gene organization and evolution [59]. This study found substantial sequence identity (69.89%) between HaKCS1-1 and AtKCS1, as well as their co-clustering in the δ-subclass, indicating functional conservation. Given that *AtKCS1* regulates cuticular wax deposition during *Arabidopsis* development [24], *HaKCS1-1* is anticipated to play a similar role in sunflower. Furthermore, phylogenetic study found that *HaKCS* genes shared homologous sequences from monocot and dicot plants across all subclasses, suggesting that *HaKCS* genes were most likely duplicated followed by subfunctionalization, which could explain why sunflower has more specialized or diverse wax-related physiological adaptations than the model plant *Arabidopsis*.

Large-scale segmental or whole-genome duplication events, rather than tandem duplication, were responsible for the majority of *HaKCS* gene expansion. This shows that polyploidization and chromosomal rearrangement played significant roles in the expansion of *HaKCS* gene family [60]. Comparative genomic research studies indicated that the *KCS* genes of sunflower, *Arabidopsis*, and safflower lacked considerable sequence homology or extensive collinearity, implying that *KCS* genes may have diverged functionally over time. Furthermore, low Ka/Ks ratios indicate that *HaKCS* genes have undergone strong purifying selection, which support the gene family’s evolutionary conservation.

### *HaKCS* genes expression were induced by ABA and responsive to drought stress

Plants respond to drought stress using two different signal transduction pathways: ABA-dependent and ABA-independent [61]. ABA is a key phytohormone that promotes cuticular wax production in flowering plants [62, 63]. Analysis of the *cis*-acting elements in promoter regions of *HaKCS* genes revealed numerous ABA-responsive elements (ABREs) and drought responsive MYC elements, indicating that these genes are likely regulated by ABA signaling and contribute to the drought responses. The tissue-specific expression patterns indicate that *HaKCS* genes are mostly active in vegetative organs, where they may regulate the structure and content of cuticular wax to maintain the integrity of the organ surface and prevent non-stomatal water loss. On the other hand, some members, like *HaKCS10-1*, show strong expression in developing seeds at 24 DAF, a time of rapid oil accumulation, suggesting a specialized role in lipid production and VLCFAs elongation during seed maturation. These results highlight the functional diversity of *HaKCS* genes and link different physiological roles in sunflower tissues to their expression levels.

Abiotic stresses such as drought, high salt, low temperature, and high temperature can all affect plant cuticular wax production [64]. In this study, we found that the majority of *HaKCS* gene expression was upregulated in stems after 1 h of drought treatment, whereas their expression was generally reduced in leaves compared to the control. In contrast, exogenous ABA treatment significantly increased the expression of most *HaKCS* genes in both tissues. This contrasting response suggests that *HaKCS* genes are differentially regulated in tissue-dependent fashion, with stems exhibiting rapid transcriptional activation that may support early reinforcement of cuticular barriers, whereas leaves exhibit a distinct regulatory pattern, possibly reflecting differences in wax deposition or physiological function.

Drought stress causes ABA accumulation and activates downstream signaling pathway that help coordinate adaptive responses [65]. *KCS* genes in *Arabidopsis* are involved in drought responses [25, 66], and that exogenous ABA can promotes cuticular wax deposition during drought stress [62]. Taken together, our findings imply that ABA signaling may function as a crucial regulator of *HaKCS* gene expression, regulating tissue-specific modifications in wax production. In response to environmental signals, such regulation probably helps to optimize cuticular features including permeability and structural organization.

### *HaKCS1-1* enhances drought stress tolerance in *Arabidopsis*

In higher plants, *KCS* genes are involved in VLCFAs synthesis and play an important role in drought response [67]. Under drought, stomatal closure and increased cuticular wax work together to reduce plant water loss [68]. Furthermore, KCS enzymes have tight substrate selectivity for carbon chain length, which influences the amount and composition of cuticular wax and increasing plant stress tolerance [69]. In sunflower seeds, *HaKCS* made the transition from 18:0-CoA to 20:0-CoA [70]. Drought increases the expression of *KCS* genes in barley, which increases the production of VLCFA and promotes the generation of cuticle wax [71].

The roles of the majority of *KCS* family members in *Arabidopsis* are unknown. Only nine of the 21 *Arabidopsis* kcs mutant lines exhibit phenotypic defects associated to wax synthesis [28, 57, 72, 73]. Similarly, heterologous expression of 18 AtKCS in yeast revealed that only nine have confirmed enzymatic activity [28, 74, 75]. The *Arabidopsis* KCS1 enzyme has a wide substrate specificity and catalyzes the synthesis of saturated and monounsaturated C16-C24 acyl-CoAs [23]. The *Arabidopsis* KCS20 and KCS2/DAISY enzymes catalyze the elongation of two-carbon units to produce C22 VLCFAs, which are necessary for the formation of cuticular wax and root suberin in the stem [9]. A functional investigation of sunflower *HaKCS1* and *HaKCS2* (corresponding to *HaKCS11-3* and *HaKCS11-2*, respectively) stimulated the elongation of C18 fatty acids in sunflower seeds, stimulate the elongation of C18 fatty acids in sunflower seeds, resulting in the production of C20-C24 fatty acids in sunflower oil [31]. Phylogenetic analysis shows that these two genes belong to the same ζ-subgroup and are closely related to *AtKCS2* and *AtKCS20* (Fig. 1). In this study, we found that *HaKCS1-1* has the highest amino acid sequence similarity to AtKCS1 while differing from other *AtKCS* genes. Furthermore, heterologous expression of *HaKCS1-1* in the *Arabidopsis kcs1* mutant significantly increased the amounts of several lipid components, including C20:0 and C32:0 fatty acids, C29:0, C31:0, and C43:0 alkanes, and C26:0 primary alcohol (Fig. 7A). Thus, we postulate that *HaKCS1-1* plays a role in C20 to C43 biosynthesis during VLCFA elongation in sunflower.

The upregulation of *HaKCS1-1* by both PEG-simulated drought and exogenous ABA indicates that it is involved in stress-responsive regulatory pathways. Our functional complementation in the *Arabidopsis kcs1* mutant shows that *HaKCS1-1* provides better drought tolerance in addition to rescuing the drought-sensitive phenotype of the *kcs1* mutant (Fig. 8C). This enhanced tolerance, as shown by lower water loss and chlorophyll leaching compared to the *kcs1* mutant, highlights the role of *HaKCS1-1* in drought response and limiting non-stomatal water loss. Furthermore, the *HaKCS1-1* overexpression lines generated much more fatty acids (C20:0 and C32:0), alkanes (C29:0, C31:0, and C43:0), and primary alcohol (C26:0) than the *kcs1* mutant. These data imply that *HaKCS1-1* may improve drought tolerance in *Arabidopsis* by modifying the composition of cuticular waxes, which in turn affects wax crystal morphology and cuticle permeability. Given the complicated evolution of sunflower *HsKCS* genes, different members are anticipated to contribute to different aspects of wax biosynthesis and stress response. Further research is needed to understand the coordinated regulation and functional specialization of *HaKCS* genes in sunflower.

## Conclusion

This study examines all 26 *HaKCS* genes in the sunflower genome, including gene structure, conserved motifs and evolutionary relationships. *HaKCS* genes showed different tissue-specific expression patterns and responded to ABA and drought treatments. Functional investigation revealed that heterologous expression of *HaKCS1-1* in the *Arabidopsis kcs1* mutant improves drought tolerance and alters the accumulation of VLCFAs and their derivatives. Collectively, these findings highlight the role of *HaKCS* genes in cuticular wax biosynthesis and stress adaptation, and identify potential targets for enhancing drought resistance in oilseed crops.

## Supplementary Information

Supplementary Material 1.

Supplementary Material 2.

Supplementary Material 3.

Supplementary Material 4.

Supplementary Material 5.

Supplementary Material 6.

## Data Availability

The Arabidopsis KCS protein sequences (AtKCS) were obtained from TAIR ( https://www.Arabidopsis.org/ ). The conserved KCS domain profiles (PF08541, 3-oxoacyl-[acyl-carrier-protein (ACP)] synthase III C-terminal; and PF08392, FAE1/type III polyketide synthase-like protein) were obtained from the Pfam protein family database ( http://www.pfam.org/ ). To identify the KCS gene family in the sunflower genome, the genome file and protein sequences were downloaded from the Ensembl Plants ( http://plants.ensembl.org/index.html ). The protein sequences of lettuce (Lactuca sativa) and maize (Zea mays) be downloaded from the NCBI database (https://static.pubmed.gov/portal/portal.fcgi/). Safflower genomic data were retrieved from https://safflower.scuec.edu.cn/.
