# Supplementary material for: Genome-wide analysis of KCS gene family in sunflower reveals the involvement of HaKCS1-1 in wax biosynthesis and drought resistance
Source: BMC Plant Biol. 2026 Jul 2;26:1626. doi: 10.1186/s12870-026-09395-9 (PMC13595779; doi:10.1186/s12870-026-09395-9)
Supplement: Supplementary file 1 [file 12870_2026_9395_MOESM1_ESM.docx]

**Table S1.** Primers used in this study.

| Primer name | Forward primer sequence | Reverse primer sequence | Primer use |
| --- | --- | --- | --- |
| *HaKCS1-1* | GCATTTCTGCATTCACGCGG | CCTTTGGCTTCCGCATACGA | qRT-PCR |
| *HaKCS1-3* | TCACCACCTTCTTGTTAGTTTCACA | GGACGTATGTCTCGTCCCCT |  |
| *HaKCS6-1* | GAACCTCCAGTTGTCCGCAG | CACCGCGCTGTTACACTTGA |  |
| *HaKCS6-2* | TTAGTCCTTCCGGCATCCGA | GAAGCTCGTCGATCACTGCC |  |
| *HaKCS5-2* | TCGCGGCCAACTCGTTAAAA | TCAATAACCGCACGTCCACC |  |
| *HaKCS9-1* | ACAAACGCCGCGCAAAATAC | TTTGCTTTTAACGCGCTGCC |  |
| *HaKCS9-2* | CTTATCCGGCCTCATCGCAC | GGATTCGTTGAACTCGCCGT |  |
| *HaKCS10-1* | ACCGAAATTGTCAACCGGGG | ACACCTCCTCCCTGCCAAAT |  |
| *HaKCS10-2* | TGACCTTTTCGGTCCGGGTA | CTCAAGCTCCCAATCTCGGC |  |
| *HaKCS10-3* | TCCACCGGAGAACAACACCT | GGCCAAATAGATCGCGTGGT |  |
| *HaKCS11-1* | TCGGTCCACTTATCGACGCT | GCGGCGAAATCCACAAGGTA |  |
| *HaKCS20-1* | AGTCATGGTGGTGCTTAGCTTG | TGACCGGTCCATGAATGTCTCT |  |
| *HaKCS20-2* | CTCACTACGCGCGATATGGTT | CGGGCTTGTAAGCGGAGAAA |  |
| *18S rRNA* | CTACCACATCCAAGGAAGGCAG | CGACAGAAGGGACGAGTAAACC |  |
| K1 | CGCGGATCCATGGATACTAATAATACGGT（*Bam*HⅠ） | GCGTCGACTTAAACCGGAACTTTAACCG（*Sal*Ⅰ） | *HaKCS1-1* cloning and *pCAMBIA2300- HaKCS1-1* construction |
| P1 | TCCCCCGGGATGGATACTAATAATACGGT（*Sam*Ⅰ） | CGCGGATCCAACCGGAACTTTAACCGGGT（*Bam*HⅠ） | *pAN580-HaKCS1-1-eGFP* vector Construction |
| AtActin | GCTCCTCTTAACCCAAAGGC | CACACCATCACCAGAATCCAGC | Semi-quantitative RT-PCR assays |
| q*HaKCS1-1* | GCATTTCTGCATTCACGCGG | CCTTTGGCTTCCGCATACGA |  |
