# Supplementary material for: Genome-wide analysis of KCS gene family in sunflower reveals the involvement of HaKCS1-1 in wax biosynthesis and drought resistance
Source: BMC Plant Biol. 2026 Jul 2;26:1626. doi: 10.1186/s12870-026-09395-9 (PMC13595779; doi:10.1186/s12870-026-09395-9)
Supplement: Supplementary file 2 [file 12870_2026_9395_MOESM2_ESM.docx]

**Table S2.** Basic information of *HaKCS* genes in sunflower.

| Gene Name | Transcript ID | Genomics position | CDS | AA size | MW (kDa) | pI | Localization prediction | Instability  index | Aliphatic  index | Grand average of  hydropathicity |
| --- | --- | --- | --- | --- | --- | --- | --- | --- | --- | --- |
| *HaKCS1-1* | OTF95193 | Chr 15: 62521609- 62524383 | 1572 | 523 | 58.44 | 9.03 | endoplasmic reticulum | 33.07 | 92.29 | 0.011 |
| *HaKCS1-2* | OTF87535 | Chr 17: 169716194- 169717688 | 1428 | 475 | 53.37 | 8.97 | nuclear | 30.30 | 99.31 | -0.002 |
| *HaKCS1-3* | OTF87559 | Chr 17: 171161029- 171162465 | 1437 | 478 | 53.63 | 8.94 | mitochondrial | 34.28 | 93.83 | 0.013 |
| *HaKCS4-1* | OTG33898 | Chr 02: 49608351-49609334 | 984 | 327 | 36.39 | 9.56 | nuclear | 26.45 | 93.27 | -0.031 |
| *HaKCS4-2* | OTG33902 | Chr 02: 49704972-49708950 | 1725 | 574 | 64.68 | 9.44 | mitochondrial | 35.49 | 92.20 | -0.057 |
| *HaKCS4-3* | OTG27902 | Chr 04: 55396018-55397529 | 1512 | 503 | 56.54 | 9.27 | endoplasmic reticulum | 36.72 | 95.13 | -0.066 |
| *HaKCS4-4* | OTG12103 | Chr 10: 194411595- 194413133 | 1539 | 512 | 57.77 | 9.13 | endoplasmic reticulum | 31.81 | 94.80 | -0.055 |
| *HaKCS5-1* | OTG07185 | Chr 11: 29023901- 29025259 | 1359 | 452 | 50.75 | 9.17 | nuclear | 38.08 | 96.44 | -0.013 |
| *HaKCS5-2* | OTF93105 | Chr 16: 171661203- 171662642 | 1488 | 479 | 53.94 | 9.1 | endoplasmic reticulum | 40.82 | 99.16 | 0.049 |
| *HaKCS5-3* | OTF87941 | Chr 17: 198222520- 198223935 | 1416 | 471 | 53.08 | 9.09 | mitochondrial | 44.99 | 97.32 | -0.014 |
| *HaKCS6-1* | OTG05699 | Chr 12: 97496384- 97497907 | 1488 | 495 | 55.63 | 9.22 | endoplasmic reticulum | 37.18 | 99.62 | 0.023 |
| *HaKCS6-2* | OTF95361 | Chr 15: 69830663- 69833188 | 1491 | 496 | 55.77 | 9.21 | endoplasmic reticulum | 37.22 | 100.04 | 0.031 |
| *HaKCS9-1* | OTG31016 | Chr 03: 71705853-71707847 | 1542 | 513 | 56.88 | 9.28 | endoplasmic reticulum | 34.56 | 91.81 | -0.048 |
| *HaKCS9-2* | OTG14490 | Chr 09: 93447100- 93449397 | 1536 | 511 | 57.00 | 9.1 | endoplasmic reticulum | 34.37 | 93.54 | -0.037 |
| *HaKCS10-1* | OTG24411 | Chr 05: 42022888- 42027773 | 1698 | 565 | 63.62 | 9.09 | endoplasmic reticulum | 46.63 | 82.32 | -0.169 |
| *HaKCS10-2* | OTG15510 | Chr 09: 155360199- 155364834 | 1650 | 549 | 61.43 | 9.34 | endoplasmic reticulum | 37.07 | 88.09 | -0.160 |
| *HaKCS10-3* | OTG12465 | Chr 10: 211909219- 211912813 | 1653 | 550 | 61.77 | 9.26 | endoplasmic reticulum | 37.36 | 87.20 | -0.162 |
| *HaKCS11-1* | OTG32993 | Chr 03: 165097234-165099656 | 1545 | 514 | 57.67 | 9.15 | endoplasmic reticulum | 38.97 | 93.72 | -0.031 |
| *HaKCS11-2* | OTG01474 | Chr 13: 95569496- 95572452 | 1545 | 514 | 57.50 | 9.19 | endoplasmic reticulum | 42.89 | 91.77 | -0.018 |
| *HaKCS11-3* | OTF91775 | Chr 16: 99694449- 99699801 | 1497 | 498 | 56.27 | 9.07 | endoplasmic reticulum | 36.85 | 98.27 | 0.035 |
| *HaKCS12-1* | OTG36655 | Chr01: 66549377-66550774 | 1398 | 465 | 52.55 | 8.09 | endoplasmic reticulum | 43.42 | 91.98 | -0.109 |
| *HaKCS12-2* | OTG21137 | Chr 07: 84190722- 84192110 | 1389 | 462 | 52.48 | 8.39 | endoplasmic reticulum | 34.56 | 89.44 | -0.162 |
| *HaKCS12-3* | OTG18919 | Chr 08: 94385316- 94386716 | 1401 | 466 | 53.05 | 8.75 | nuclear | 40.08 | 86.20 | -0.214 |
| *HaKCS19* | OTF99522 | Chr 14: 162618768- 162620440 | 1365 | 454 | 51.43 | 8.62 | endoplasmic reticulum | 41.00 | 91.92 | -0.115 |
| *HaKCS20-1* | OTG03395 | Chr 13: 187916136- 187918713 | 1482 | 493 | 55.92 | 9.3 | endoplasmic reticulum | 32.37 | 95.13 | -0.061 |
| *HaKCS20-2* | OTG03396 | Chr 13: 187919340- 187923678 | 1542 | 513 | 57.75 | 9.19 | endoplasmic reticulum | 36.79 | 94.11 | -0.038 |
