# Supplementary material for: Genome-wide analysis of KCS gene family in sunflower reveals the involvement of HaKCS1-1 in wax biosynthesis and drought resistance
Source: BMC Plant Biol. 2026 Jul 2;26:1626. doi: 10.1186/s12870-026-09395-9 (PMC13595779; doi:10.1186/s12870-026-09395-9)
Supplement: Supplementary file 3 [file 12870_2026_9395_MOESM4_ESM.docx]

**Table S4.** Putative motifs identified from HaKCS proteins using MEME. The sequence logos were generated using WebLogo.

| **Motif name** | **Sequence logo** |
| --- | --- |
| **Motif 1** |   RMTLHRFGNTSSSSJWYELAYMEAKGRVKKGDRVWQIAFGSGFKCNSAVW |
| **Motif 2** |   DIGILVVNCSLFNPTPSLSSMIVNHYKLRGNIKSFNLGGMGCSAGLIAID |
| **Motif 3** |   NWYFGNERSMLJPNCLFRMGGAAVLLSNKSSDRRRAKYKLVHVVRTHKGA |
| **Motif 4** |   YNCVYQEEDDEGKIGVSLSKDLMAIAGDALKTNITTLGPLVLPISEQLLF |
| **Motif 5** |   KPYIPDFKLAFEHFCIHAGGRAVIDELZKNLQLSDEHVEPS |
| **Motif 6** |   EFQKKILERSGLGEETYLPKAI |
| **Motif 7** |   PPNPSMAEARAEAEMVMFGAJDDLFAKTG |
| **Motif 8** |   LAKDLLQVHPNSYAVVVSTEN |
| **Motif 9** |   FLSTVYFMSRPRPVYLVDFACYKPPD |
| **Motif 10** |   DFLQSVKLKYVKLGYHYLISHGLYLLLVP |
