# Supplementary material for: Genome-wide analysis of KCS gene family in sunflower reveals the involvement of HaKCS1-1 in wax biosynthesis and drought resistance
Source: BMC Plant Biol. 2026 Jul 2;26:1626. doi: 10.1186/s12870-026-09395-9 (PMC13595779; doi:10.1186/s12870-026-09395-9)
Supplement: Supplementary file 4 [file 12870_2026_9395_MOESM5_ESM.docx]

**Table S5.** The Ka, Ks, Ka/Ks ratio and divergence time of duplicate *HaKCS* genes in sunflower.

| Duplicated gene pairs | Ka | Ks | Ka/Ks | Duplicated type | Selective pressure | Divergence Time (Mya) |
| --- | --- | --- | --- | --- | --- | --- |
| *HaKCS10-2/HaKCS10-3* | 0.0501 | 0.7788 | 0.0644 | Segmental | Purifying selection | 25.9600 |
| *HaKCS11-3/HaKCS20-2* | 0.0991 | 1.6379 | 0.0605 | Segmental | Purifying selection | 54.5967 |
| *HaKCS11-3/HaKCS20-1* | 0.1255 | 1.7622 | 0.0712 | Segmental | Purifying selection | 58.7400 |
| *HaKCS5-2*/*HaKCS5-3* | 0.0848 | 0.6870 | 0.1234 | Segmental | Purifying selection | 22.9000 |
| *HaKCS6-1/HaKCS6-2* | 0.0751 | 1.1683 | 0.0643 | Segmental | Purifying selection | 38.9433 |
| *HaKCS11-1/HaKCS20-2* | 0.0576 | 0.9684 | 0.0595 | Segmental | Purifying selection | 32.2800 |
| *HaKCS11-1/HaKCS20-1* | 0.0797 | 0.9815 | 0.0812 | Segmental | Purifying selection | 32.7167 |
| *HaKCS20-1/HaKCS20-2* | 0.0614 | 0.4198 | 0.1462 | Tandem | Purifying selection | 13.9933 |
