# Supplementary material for: Genome-wide analysis of KCS gene family in sunflower reveals the involvement of HaKCS1-1 in wax biosynthesis and drought resistance
Source: BMC Plant Biol. 2026 Jul 2;26:1626. doi: 10.1186/s12870-026-09395-9 (PMC13595779; doi:10.1186/s12870-026-09395-9)
Supplement: Supplementary file 5 [file 12870_2026_9395_MOESM6_ESM.docx]

**Fig. S1.** Generation and molecular characterization of transgenic *Arabidopsis* plants. (A) Expression detection of *HaKCS1-1* gene in transgenic plant; M: DL2000 Plus DNA Marker; 1-3: *HaKCS1-1/kcs1*; 4-5: *kcs1*; 6, 8, 10: *kcs1*; 7, 9, 11: *HaKCS1-1/kcs1.* The band sizes of *Ataction* and *HaKCS1-1* qRT-PCR were 165 bp and 156 bp respectively. (B) 1, 2, 3, 4: Identification of transgenic *Arabidopsis*; *kcs1*: *Arabidopsis kcs1* mutants; *HaKCS1-1/ kcs1*: *HaKCS1-1* gene overexpression in *Arabidopsis kcs1* mutant. The total length of *HaKCS1-1* CDS is 1572 bp.
